# Med-Diet: evaluation of an LLM-based system for clinically guided nutrition care in chronic diseases

**DOI:** 10.3389/fnut.2026.1826469

**Published:** 2026-06-12

**Authors:** Yanan Wang, Miaomiao Cheng, Qi Zhang, Jian Feng, Jun Qin, Lei Gong, Lei Liu, Yan Zhao, Shasha Zhao, Fanli Meng, Longyan Chen, Tingjun Dai, Pengfei Lin, Jie Peng, Yun Ti, Lijun Song, Qi Liu, Hua Liang, Xiaoyan Xiao

**Affiliations:** 1 Department of Nephrology, Qilu Hospital of Shandong University, Jinan, Shandong, China; 2 Healthcare Big Data Research Institute, Cheeloo College of Medicine, Shandong University, Jinan, Shandong, China; 3 Department of Clinical Nutrition, Qilu Hospital of Shandong University, Jinan, Shandong, China; 4 Department of Endocrinology and Metabolism, Qilu Hospital of Shandong University, Jinan, Shandong, China; 5 Department of Hepatology, Qilu Hospital of Shandong University, Jinan, Shandong, China; 6 Department of Neurology, Qilu Hospital of Shandong University, Jinan, Shandong, China; 7 Department of Cardiology, Qilu Hospital of Shandong University, Jinan, Shandong, China; 8 Department of Rheumatology, Qilu Hospital of Shandong University, Jinan, Shandong, China

**Keywords:** AI-based dietary plan, clinical nutrition, dietary management, large language models, noncommunicable chronic diseases

## Abstract

**Background:**

Scientifically grounded and clinically applicable dietary management is essential for patients with chronic diseases. However, in routine practice, nutritionists frequently lack efficient and scalable tools to deliver targeted, guideline-consistent nutritional guidance across diverse and complex clinical scenarios.

**Objective:**

To develop and conduct an exploratory expert-rating evaluation of Med-Diet, a large language model (LLM)—based agent for generating dietary plans for chronic diseases.

**Methods:**

We built Med-Diet using DeepSeek-R1, integrated clinical dietary guidelines, evaluated it on 79 real cases covering common, rare, and complex noncommunicable diseases, and compared it with four general-purpose LLMs (DeepSeek-R1, GPT-4o, GLM-Z1-32B, and Llama-3.3-70B). Fourteen clinical experts from different fields conducted blinded, multidimensional ratings of generated dietary plans. Furthermore, an exploratory comparative experiment assessed nutritionists’ efficiency and output quality without and with Med-Diet assistance.

**Results:**

Med-Diet received higher mean preference scores from expert evaluators compared to all baseline LLMs (mean score of 4.09 ± 0.64). Expert ratings suggested superior performance for Med-Diet in dimensions including accuracy, safety, nutritional balance, personalization, practicality, and overall recommendation (all *p* < 0.05). DeepSeek-R1 ranked second with an overall average score of 3.60 ± 0.68. This model performed the strongest in rare disease scenarios but lagged behind Med-Diet in common diseases and complex cases. GPT-4o (3.33 ± 0.66) and GLM-Z1-32B (3.35 ± 0.75) showed moderate and inconsistent performance, while Llama-3.3-70B performed the worst (3.00 ± 0.69). When nutritionists used Med-Diet to assist in dietary plan generation, the median time required decreased from 17.5 min to 13.0 min (*p* < 0.05). Expert scores for accuracy, personalization, practicality, and overall recommendation were higher in the Med-Diet-assisted group (adjusted *p* < 0.05).

**Conclusion:**

In this expert-rating study, Med-Diet-generated dietary plans received higher preference scores from clinical experts compared to those from general-purpose LLMs. These preliminary findings suggest that knowledge injection and framework constraints of Med-Diet may improve expert-perceived quality of AI-generated dietary plans. Med-Diet shows potential as an adjunctive tool in the dietary management of chronic diseases, but its clinical safety and effectiveness require prospective validation.

## 1 Introduction

Noncommunicable diseases (NCDs), including cardiovascular disease, chronic kidney disease (CKD), and diabetes, remain major global health challenges and account for the majority of premature mortality worldwide (1). The treatment and prevention of these chronic conditions constitute a crucial challenge for public health. Unhealthy dietary habits serve as a modifiable factor that play a driving role in the development of various diseases (2). Therefore, medical nutrition therapy plays a vital role in disease control, complication prevention, and quality-of-life improvement (3, 4).

In the healthcare field, recent advancements in AI, particularly the application of large language models (LLMs), have demonstrated significant potential in supporting health communication, medical diagnosis, medical record documentation, and patient education (5–7). Since the release of LLMs, numerous nutrition management studies have developed or employed LLM-based chatbots targeting different health conditions, such as cardiovascular diseases, type 2 diabetes, and chronic kidney disease (8, 9). The primary applications in the nutrition field include personalized nutrition (10, 11), food intake tracking and recognition (12), dietary assessment (13), and predictive analytics for disease prevention and treatment (14).

Despite growing research in AI-assisted nutrition, systematic evaluations of whether LLMs can generate accurate, safe, and actionable dietary plans across diverse clinical scenarios remain limited. To address these gaps, we developed Med-Diet, a clinical nutrition AI assistant enhanced with domain knowledge. To preliminarily explore the potential utility of LLMs in personalized nutritional care from an expert evaluation perspective. This study compared Med-Diet with general-purpose LLMs across diverse clinical scenarios and examined its impact on efficiency and output quality within a small-scale, exploratory nutritionist-AI collaborative workflow.

## 2 Methods

### 2.1 Ethical considerations

This study included a total of 79 real-world patient electronic medical records. The study obtained approval from the Medical Ethics Committee of Qilu Hospital of Shandong University (approval number: KYLL-202506-007-1).

### 2.2 Development of Med-Diet

**Figure 1:**
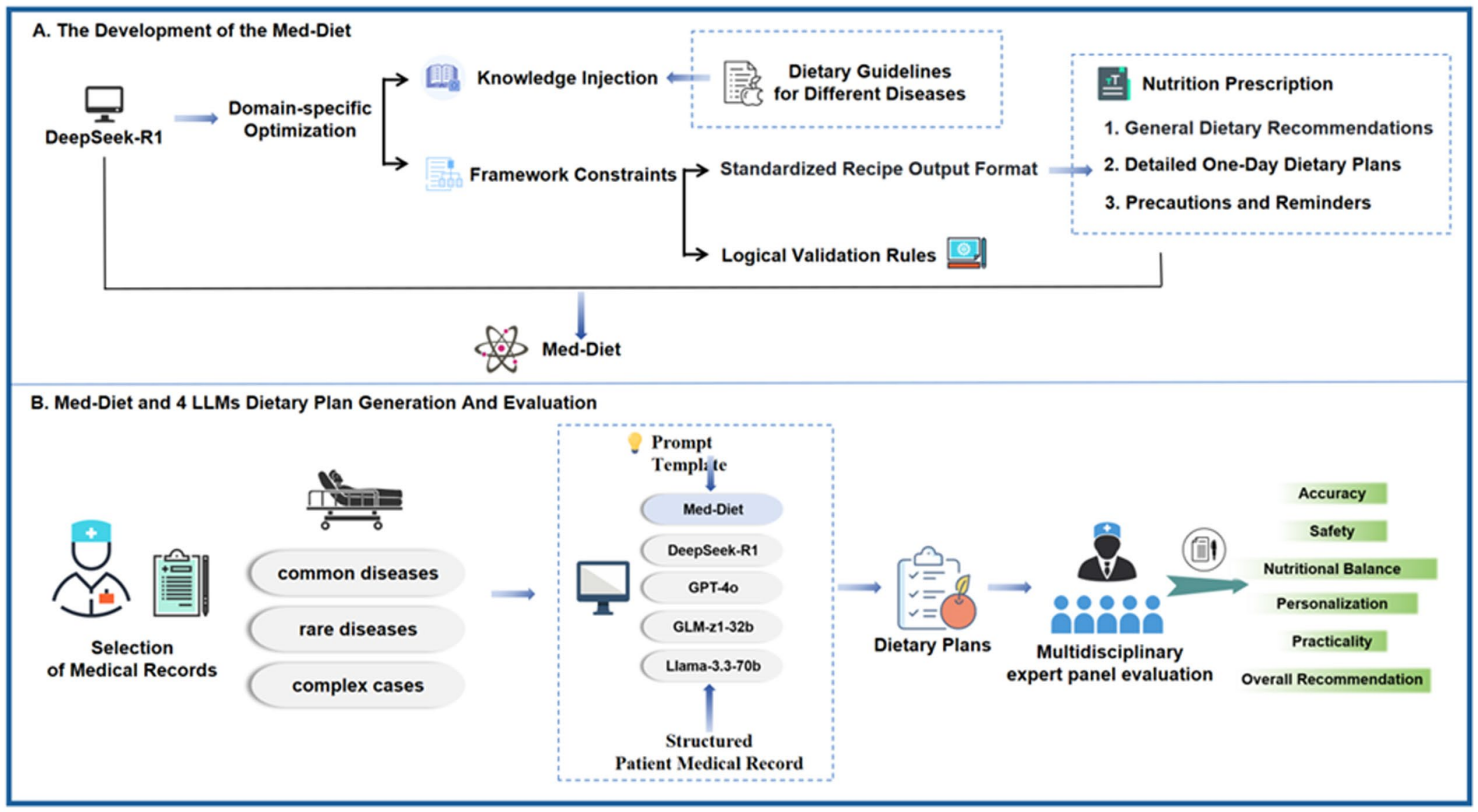
The Med-Diet system architecture and operational workflow. **(A)** Med-Diet is built by optimizing DeepSeek-R1 through two core approaches: knowledge injection (integration of clinical dietary guidelines) and framework constraints (a customized dietary plan template). **(B)** A structured prompt template providing the same instructions and patient information to all evaluation models.

Med-Diet was developed based on the DeepSeek-R1 model. We performed domain-specific optimization through two core approaches: knowledge injection (integrating authoritative dietary guidelines for 17 diseases, e.g., ADA, ESPEN, KDIGO, Chinese Medical Association consensus, etc.) and framework constraints (a customized dietary plan framework), thereby constructing the Med-Diet agent (Figure 1A). The operational workflow of Med-Diet follows a two-stage reasoning process designed to enhance safety and personalization. First, a disease diagnosis module analyzes the patients’ structured information (e.g., demographics, lab results) against a predefined knowledge base of diagnostic criteria to identify all relevant conditions. The output of this stage is a structured list of confirmed diagnoses. Second, a dietary prescription module integrates the confirmed diagnoses, together with the patient’s individual profile (e.g., dietary preferences and allergies). This module is constrained by a standardized output template that mandates a comprehensive dietary plan, including overall principles, a detailed daily plan, and key safety precautions. This approach ensures that the final output complies with medical guidelines while being tailored to the individual patient. The detailed, structured prompts used for the disease diagnosis and dietary prescription modules of Med-Diet are presented in Supplementary material S1.

### 2.3 Selection of general-purpose LLMs

To ensure a comprehensive and fair evaluation of Med-Diet’s performance, we selected four LLMs that are representative in terms of model capability, openness, and real-world influence as baseline comparators. All models were invoked via their official application programming interfaces (APIs) or corresponding open-source versions.

DeepSeek-R1 is a general-purpose LLM with strong logical reasoning and long-form text generation capabilities. Additionally, as the base model of Med-Diet, it serves as a reference for evaluating Med-Diet’s performance. GPT-4o (OpenAI), a closed-source commercial LLM with advanced multimodal capabilities and strong language understanding and generation performance, was included as a benchmark for state-of-the-art proprietary models. GLM-Z1-32B (Zhipu AI), a bilingual model with demonstrated strength in Chinese-language contexts, was selected to assess performance in Chinese clinical scenarios. Llama-3.3-70B (Meta) is a widely used open-source large-parameter language model with strong language processing capabilities and learning potential, capable of handling more complex and diverse tasks.

### 2.4 Case selection and data preprocessing

All case information in this study was obtained from authentic, de-identified electronic medical records at Qilu Hospital of Shandong University. These records were reviewed by specialists from the relevant clinical departments to ensure clinical validity. The cases spanned seven major clinical systems (including Cardiology, Nephrology, Endocrinology, Hepatology, Neurology, Rheumatology, and Hematology), with a total of 79 cases included. The cases were categorized into three groups to reflect varying levels of clinical complexity and representativeness: (1) Common Diseases Group (*n* = 70): This group covered 14 prevalent conditions, including coronary heart disease, hyperlipidemia, nephrotic syndrome, chronic kidney disease, dialysis, diabetes, hyperthyroidism, osteoporosis, obesity, liver cirrhosis, cerebrovascular accident, systemic lupus erythematosus, gout, and iron deficiency anemia. For each disease, five patients with different clinical manifestations were selected to evaluate the model’s ability to apply basic nutritional principles. (2) Rare Disease Group (*n* = 3): This group included 3 conditions—phenylketonuria, methylmalonic acidemia, and Wilson’s disease. Given the extreme rarity of these diseases and the limited availability of cases, this group was only used to explore the model’s professional capabilities and its ability to handle long-tail knowledge. (3) Complex Case Group (*n* = 6): This group included 6 patients with two or more coexisting diseases involving potentially conflicting nutritional management principles (e.g., diabetes, chronic kidney disease, and heart failure). This scenario was used to explore the model’s ability to make comprehensive decisions in complex clinical conditions.

The 79 electronic medical records included complete demographic information (e.g., sex, age, ethnicity, height, weight, and body mass index), disease diagnoses and staging, key laboratory parameters, current medication regimens, as well as documented food allergies and dietary preferences. All records were anonymized prior to analysis.

### 2.5 Model input prompt design

To ensure fairness and consistency in evaluation, a structured prompt template was designed to provide identical instructions to all evaluated models. The prompt template was used to input patient information into each LLM, and the generated dietary plans were recorded for subsequent evaluation (Figure 1B).

The instructions are as follows:

You are a professional clinical nutritionist. Develop a personalized dietary plan for the patient based on the following patient information. Patient details are as follows:

- Basic Information: [Gender], [Age], [Region], [Height] cm, [Weight] kg, [BMI] kg/m^2^
- Clinical diagnosis: [e.g., type 2 diabetes]—Key biochemical indicators: [e.g., fasting blood glucose 8.5 mmol/L, serum potassium 5.1 mmol/L]
- Current medications: [e.g., metformin]
- Food allergies/intolerances: [e.g., seafood allergies]—Dietary preferences and cultural background: [e.g., preference for lighter flavors, Hui, avoid pork]
- Other relevant factors: [e.g., activity level, economy, and cooking conditions]

Develop a daily dietary plan for the patient based on the patient’s case information and individual circumstances (e.g., age, BMI, dietary preferences, clinical diagnosis, laboratory indicators). Provide total calorie and macronutrient content.

### 2.6 Nutritionist dietary plan generation and modification

**Figure 2:**
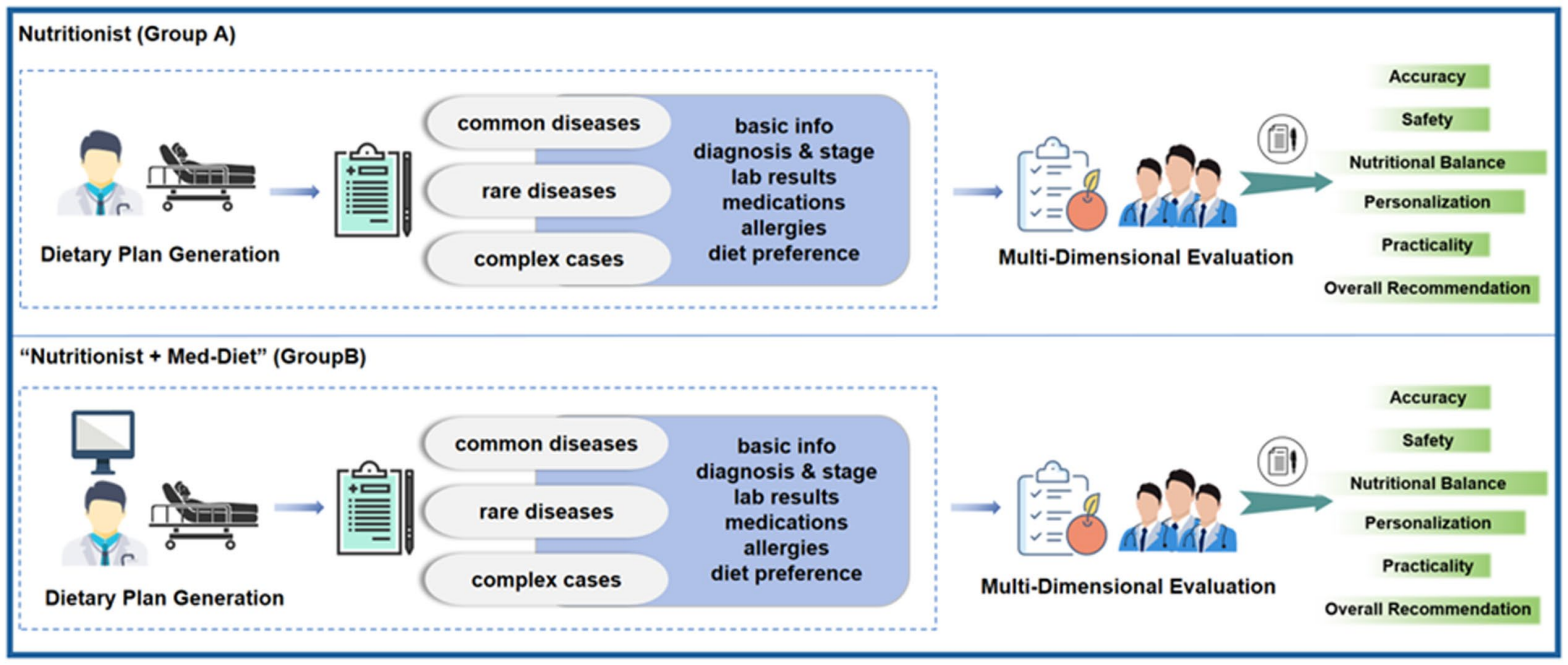
Comparison of dietary plans generation: “Nutritionist” vs. “Nutritionist + Med-Diet.” Group A (Nutritionist) independently formulated dietary plans. Group B (Nutritionist + Med-Diet) reviewed and modified dietary plans based on the model outputs. The time required to complete the final dietary plan was recorded. The dietary plans were evaluated multi-dimensionally by an expert panel.

To preliminarily assess the potential value of Med-Diet as an assistive tool in real-world workflows, we conducted an exploratory experiment (Figure 2). Three medical records were randomly selected from each of the common diseases group, rare diseases group, and complex case groups, resulting in a total of 9 patient records. Two registered nutritionists with over 5 years of clinical experience participated in the experiment. In order to minimize the learning effect of nutritionists, the time to generate dietary plans for Group A and Group B would be separated by 1 week.

Group A (Nutritionist): Two nutritionists independently generated dietary plans for the nine selected patient records without access to any model-generated outputs. The total time required, from initial review of the medical records to finalizing the dietary plan, was recorded.

Group B (Nutritionist + Med-Diet): The nutritionists used the Med-Diet to generate dietary plans for the patients and could make necessary adjustments to the resulting drafts. We recorded the total duration from the start of inputting prompts to the model to the final dietary plan. This time also included the time taken for the nutritionist to review the medical records.

### 2.7 Evaluation

#### 2.7.1 Dimensions of evaluation

An expert evaluation panel evaluated all dietary plans generated by the models using a Likert scale (1 = Very Poor, 2 = Poor, 3 = Fair, 4 = Good, 5 = Excellent). Key dimensions included: (1) Accuracy: Consistency with clinical nutrition guidelines and principles for the disease. (2) Safety: Whether the dietary plan avoids contraindicated foods that could worsen the patient’s condition or cause drug-food interactions. (3) Nutritional Balance: Whether the composition of nutrients is reasonable and comprehensive, meets disease-specific requirements, and offers a variety of food choices. (4) Personalization: Whether the dietary plan sufficiently considers the patient’s individual taste preferences and cultural or religious dietary restrictions. (5) Practicality: Whether the generated dietary plan is clear and specific, including the availability of ingredients, feasibility of cooking steps, and clarity of portion sizes. (6) Overall Recommendation: Whether the experts are willing to recommend the dietary plan to the patient after comprehensive consideration. See Supplementary Table S2 for detailed scoring.

For the two groups of dietary plans—Group A (Nutritionist) and Group B (Nutritionist + Med-Diet)—three clinical experts evaluated them across 6 dimensions: accuracy, safety, nutritional balance, personalization, practicality, and overall recommendation. This evaluation compared the differences in the final outputs of the two groups.

#### 2.7.2 Evaluation method

The dietary plans generated by the five models were anonymized and labeled as dietary plan 1 to dietary plan 5, ensuring that no expert reviewers could identify the source of each dietary plan.

This study employed a single-blind design to minimize assessment bias. We assembled a review panel comprising 12 medical specialists (covering cardiology, nephrology, endocrinology, hepatology, neurology, and rheumatology) and two nutrition specialists. Each expert evaluated all dietary plans within their field according to the assessment criteria. To ensure professionalism and reliability, each dietary plan was independently evaluated by two experts from the same field. The two nutrition experts evaluated the same dietary plan for each disease to provide an interdisciplinary perspective. Additionally, the dietary plans from Group A and Group B were evaluated using the same dimensions by three clinical experts.

### 2.8 Statistical analysis

Inter-rater reliability: The ICC (2, 1) absolute agreement (two-way random effects model) was used to assess agreement among raters. Due to small sample sizes, ICC analyses were performed only for common diseases (*n* = 70). Because experts from different specialties may have systematically different criteria for evaluating dietary plans, department-specific ICCs were calculated separately for each specialty to assess inter-rater agreement within clinical domains. The small sample sizes of rare diseases (*n* = 3) and complex cases (*n* = 6) precluded stable ICC estimation. Overall ICCs for rare and complex cases were reported only for exploratory purposes. 95% confidence intervals for ICCs were approximated using the F-distribution.

The data had a clustered structure. Multiple model outputs were generated for the same case, and multiple ratings were contributed by the same experts. Therefore, we used linear mixed models (LMMs) as the primary analytic approach. For the full dataset and the common disease subgroup, the model was included as a fixed effect, with case and rater as random intercepts. For rare diseases and complex cases, only the rater random intercept was included (case random effect not estimated). The unit of analysis was each individual score of a dietary plan on a single dimension. Likert scale scores were treated as approximate continuous variables, given the large sample size and the central limit theorem. Likelihood ratio tests (LRT) assessed overall model differences; when significant, estimated marginal means (EMMs) with Tukey adjustment were used for pairwise comparisons. Additionally, as a sensitivity analysis, we re-analyzed the data using an ordinal mixed-effects model.

Given the small sample size (*n* = 9) and the potential non-normality of the generation time data, we used the Wilcoxon signed-rank test to compare the differences in generation time between Group A and Group B. Times are reported as median (interquartile range). For the analysis of dietary plan quality, we first calculated the average score of each dietary plan across the six dimensions, as assigned by the three experts. Using the “nutritionist-case” pair as the unit of analysis, the paired Wilcoxon signed-rank test was applied to compare scores between Group A and Group B for each of the six dimensions. To control for multiple comparisons, the Bonferroni correction was applied (adjusted *α* = 0.05/6 ≈ 0.0083). All tests were two-sided with a significance level of *α* = 0.05. Effect size *r* was calculated as Z/√N.

Statistical analyses were performed using R software (version 4.4.3).

## 3 Results

### 3.1 Inter-rater reliability

The overall ICC (2, 1) for common diseases was 0.29 (95% CI: 0.26–0.31), for rare diseases 0.17 (95% CI: 0.12–0.25), and for complex cases 0.12 (95% CI: 0.05–0.19). Department-specific ICCs ranged from 0.14 (Neurology) to 0.37 (Hepatology) (Supplementary Tables S3). These values indicate a limited level of consistency among raters, reflecting the inherent limitations of subjective rating.

### 3.2 Overall performance comparison

**Figure 3:**
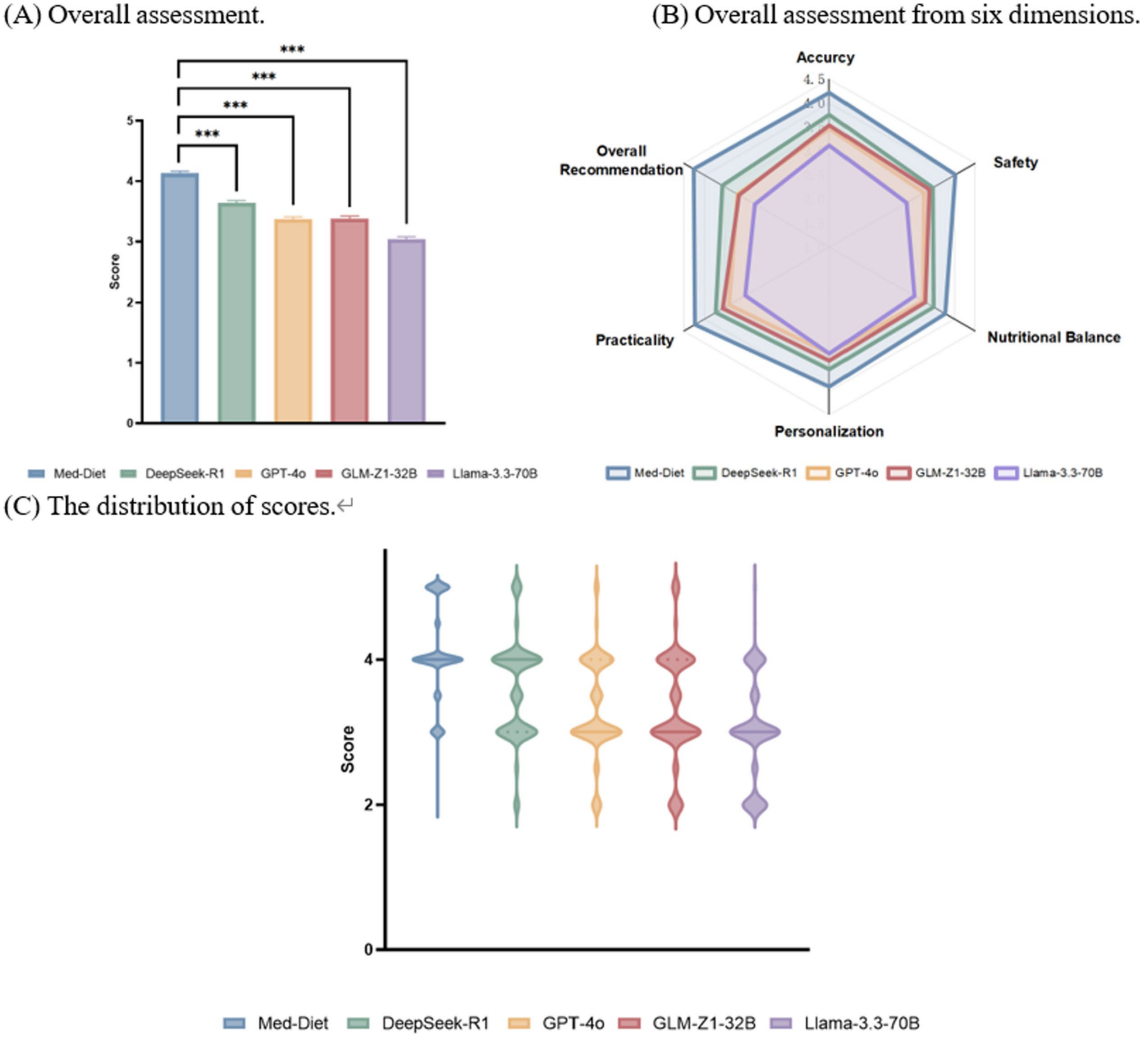
Overall performance comparison between Med-Diet and general-purpose LLMs. **(A)** Overall assessment. **(B)** Overall assessment across six dimensions. **(C)** Degree of variation in scores of each model. Significance annotations: Asterisks indicate the level of statistical significance for comparisons with Med-Diet, based on linear mixed models (LMM) with Tukey adjustment for multiple comparisons. The analysis used estimated marginal means (EMM) from the LMM (random intercepts: case and rater). *p < 0.05, **p < 0.01, ***p < 0.001, ****p < 0.0001. Boxplots and violin plots display raw data.

Across all evaluation scenarios encompassing common diseases, rare diseases, and complex cases, Med-Diet demonstrated superior performance compared to all baseline large language models (LLMs). Med-Diet achieved the highest mean score (4.09 ± 0.64), followed by DeepSeek-R1 (3.60 ± 0.68), GPT-4o (3.33 ± 0.66), GLM-Z1-32B (3.35 ± 0.75), and Llama-3.3-70B (3.00 ± 0.69). Linear mixed model (LMM) analysis confirmed significant overall differences among models (*F* (4, 6120) = 503.93, *p* < 0.05). Estimated marginal means (EMMs) and Tukey-adjusted pairwise comparisons are summarized in Table 1. As shown in Figure 3A, Med-Diet (EMM = 4.13, 95% CI = 4.02–4.24) significantly outperformed all other models (all *p* < 0.05). DeepSeek-R1 (EMM = 3.64) ranked second and was superior to GPT-4o (EMM = 3.37, *p* < 0.05) and GLM-Z1-32B (EMM = 3.38, *p* < 0.05). Notably, no significant difference was detected between GLM-Z1-32B and GPT-4o (*p* = 0.98). Llama-3.3-70B (EMM = 3.04) scored lowest and was significantly worse than all other models (all *p* < 0.05).

**Table 1:** Estimated marginal means and pairwise comparisons from the linear mixed model.

Models	EMM (95%CI)	Standard Error (SE)	Difference vs. Med-Diet (95% CI)	Difference SE	*z*-value	Adjusted *p*-value
Med-Diet	4.13 (4.02–4.24)	0.05	—	—	—	—
DeepSeek-R1	3.64 (3.53–3.75)	0.05	−0.50 (−0.44 to −0.55)	0.03	19.37	<0.001
GLM-Z1-32B	3.37 (3.26–3.48)	0.05	−0.76 (−0.71 to −0.81)	0.03	29.85	<0.001
GPT-4o	3.38 (3.27–3.49)	0.05	−0.75 (−0.70 to −0.80)	0.03	29.27	<0.001
Llama-3.3-70B	3.04 (2.93–3.15)	0.05	−1.09 (−1.04 to −1.14)	0.03	42.68	<0.001

Pairwise comparisons used Tukey’s adjustment; all comparisons based on LMM (random intercepts: case, rater).

Dimension-specific analyses further elucidated Med-Diet’s comprehensive advantages. Among the six evaluation dimensions (Figure 3B), Med-Diet obtained the highest mean scores in all dimensions, particularly in accuracy (4.22 ± 0.56), practicality (4.23 ± 0.62) and overall recommendation (4.26 ± 0.56). Its scores for nutritional balance (3.78 ± 0.55) and personalization (3.91 ± 0.75) were relatively lower but still significantly higher than those of other models. In addition, LMMs for each dimension revealed significant model effects for every dimension (all *p* < 0.05). Post-hoc comparisons showed that Med-Diet was significantly superior to the other four models in every dimension (all *p* < 0.05). DeepSeek-R1 ranked second. It was significantly better than GPT-4o and GLM-Z1-32B in all dimensions except safety (all *p* < 0.05), with no significant difference from GPT-4o in safety (*p* > 0.05). GPT-4o and GLM-Z1-32B performed similarly in all dimensions, and there was no statistical difference between the two. Llama-3.3-70B performed the worst, indicating highly inconsistent performance in personalization.

In terms of effect sizes, Med-Diet’s advantage over DeepSeek-R1 was largest in overall recommendation (difference 0.70) and practicality (0.48), and smallest in personalization (0.39). Absolute scores for nutritional balance were lower than those for other dimensions across all models, suggesting this dimension is a common weakness of current LLMs.

Figure 3C shows the score distribution of the five models. Med-Diet had the highest central tendency (median = 4) and the smallest IQR of 0.5 (Q3–Q1 = 4.5–4). It tended to receive relatively higher scores. DeepSeek-R1 followed with a median of 4 and an IQR of 1 (Q3–Q1 = 4–3), reflecting occasional lower scores that pull the average down. GPT-4o and GLM-Z1-32B had the same medians (both 3), with the same IQR of 1 (Q3–Q1 = 4–3). But their violin shapes differ slightly: GPT-4o’s distribution appears more concentrated around the median with a modest upper tail, while GLM-Z1-32B showed a slightly broader spread and a hint of bimodality, indicating greater variability in specific evaluation contexts. Llama-3.3-70B ranked lowest (median 3, IQR = 1 from 3.5 to 2.5), with a flatter violin contour and lower tail. This indicated greater score variability compared to other models, suggesting less stable performance.

Additionally, we conducted sensitivity analyses using ordinal mixed-effects models to account for the ordinal nature of the Likert scale scores. The results of these sensitivity analyses were consistent with those obtained from the linear mixed models (Supplementary Tables S4), confirming the robustness of the primary analysis.

### 3.3 Model performance across different diseases types

#### 3.3.1 Performance on common diseases

**Figure 4:**
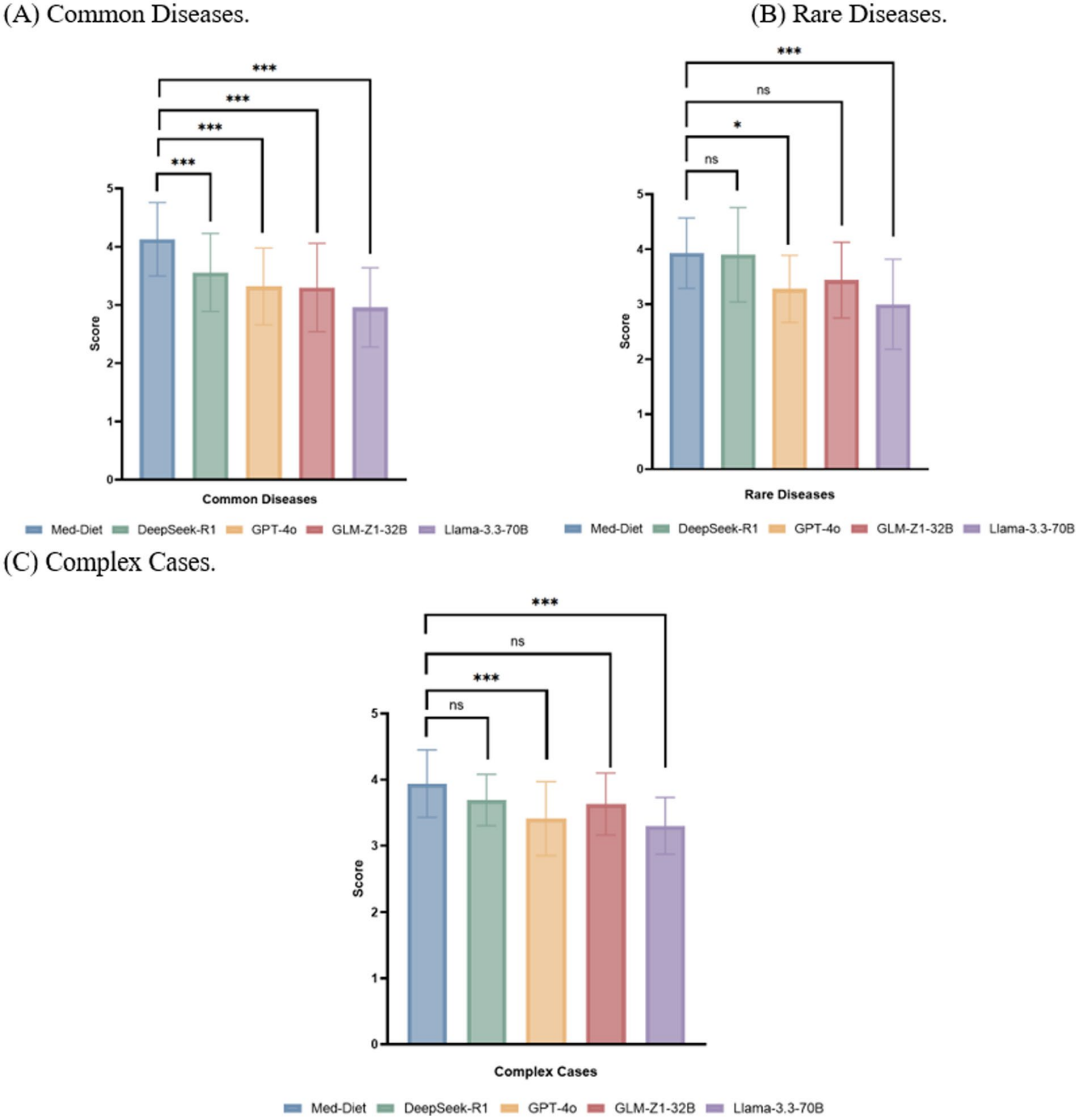
Model performance across various disease types: **(A)** Common diseases; **(B)** Rare diseases; **(C)** Complex cases. Bars represent mean raw scores (± SD). Significance asterisks indicate pairwise comparisons with Med-Diet (reference) using linear mixed models with Tukey adjustment. *p < 0.05, **p < 0.01, ***p < 0.001, ****p < 0.0001; “ns” denotes non-significant (*p* ≥ 0.05). Due to small sample sizes, results for rare diseases and complex cases are exploratory.

For common diseases, Med-Diet showed the highest mean score (4.13 ± 0.63), followed by DeepSeek-R1 (3.56 ± 0.67), GPT-4o (3.32 ± 0.66), GLM-Z1-32B (3.30 ± 0.76) and Llama-3.3-70B (2.96 ± 0.68). The LMM demonstrated a significant model effect (*F* (4, 3000) = 476.02, *p* < 0.05). Pairwise comparisons with Tukey adjustment confirmed that Med-Diet was superior to all other models (all *p* < 0.05) (Figure 4A). DeepSeek-R1 significantly outperformed GPT-4o (*p* < 0.05) and GLM-Z1-32B (*p* < 0.05), while GLM-Z1-32B and GPT-4o were not significantly different (*p* = 0.903). Llama-3.3-70B was significantly worse than all others (all *p* < 0.05).

**Figure 5:**
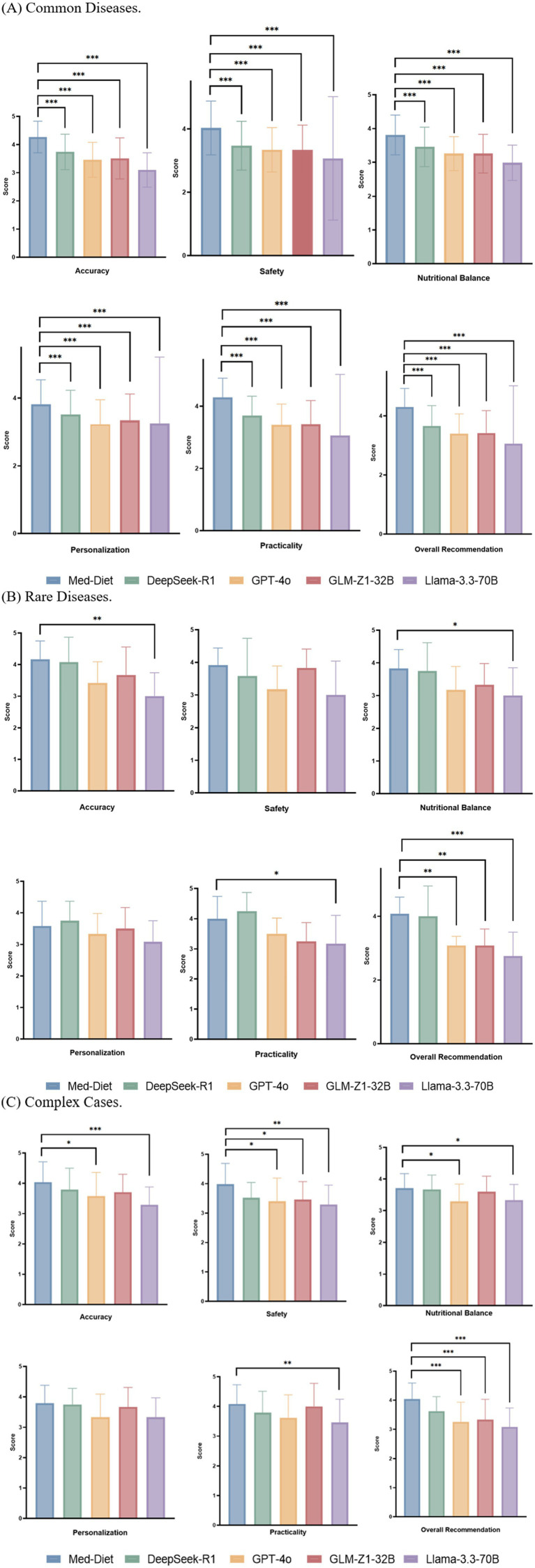
Model performance across dimensions in different disease types. **(A)** Common diseases. **(B)** Rare diseases. **(C)** Complex cases. Each panel shows mean raw scores (± SD) for the five models across the six evaluation dimensions (accuracy, safety, nutritional balance, personalization, practicality, overall recommendation). Significance asterisks indicate pairwise comparisons with Med-Diet (reference) using linear mixed models (LMM) with Tukey adjustment. The analysis used estimated marginal means (EMM) from LMM (random intercepts: case and rater). *p < 0.05, **p < 0.01, ***p < 0.001, ****p < 0.0001; “ns” denotes non-significant (*p* ≥ 0.05). Due to small sample sizes, results for rare diseases and complex cases are exploratory.

Common diseases showed different performance across various dimensions. Med-Diet demonstrated consistent superiority over general-purpose LLMs across all six dimensions in common diseases (all *p* < 0.05) (Figure 5A). Med-Diet excelled particularly in accuracy (4.27 ± 0.56 vs. 3.74 ± 0.63 for DeepSeek-R1), safety (4.03 ± 0.85 vs. 3.47 ± 0.77), and practicality (4.28 ± 0.62 vs. 3.70 ± 0.62). Notably, all models exhibited relatively lower performance in nutritional balance, with Med-Diet scoring 3.81 ± 0.59 compared to DeepSeek-R1’s 3.46 ± 0.58 and GPT-4o’s 3.26 ± 0.50. This suggests that there are inherent challenges in optimizing dietary diversity within disease-specific constraints. Personalization scores highlighted Med-Diet’s advantage (3.82 ± 0.72) over general models, which clustered between 3.22 and 3.52, indicating limitations in adapting to individual patient preferences and cultural dietary restrictions among baseline LLMs.

#### 3.3.2 Performance on rare diseases

In rare disease scenarios (phenylketonuria, methylmalonic acidemia, and Wilson’s disease), the LMM showed a significant overall model effect (*F* (4, 344) = 25.40, *p* < 0.05). Med-Diet maintained a high mean score (3.93 ± 0.64), but DeepSeek-R1 demonstrated comparable performance (3.90 ± 0.86) with no statistically significant difference between the two (*p* > 0.05) (Figure 4B). Both models significantly outperformed GPT-4o (3.28 ± 0.61) and Llama-3.3-70B (3.00 ± 0.82) (all *p* < 0.05). GLM-Z1-32B (3.44 ± 0.43) did not differ significantly from Med-Diet (*p* > 0.05) or DeepSeek-R1 (*p* > 0.05). GPT-4o, GLM-Z1-32B, and Llama-3.3-70B performed similarly, with significant differences observed only between the latter two (*p* < 0.05).

Dimension-specific analysis revealed that Med-Diet and DeepSeek-R1 performed comparably across all six dimensions, with no significant differences observed (all adjusted *p* > 0.05) (Figure 5B). Med-Diet and DeepSeek-R1 performed comparably, with both achieving high scores in accuracy (4.17 ± 0.58 and 4.08 ± 0.79, respectively), practicality (4.00 ± 0.58 and 4.25 ± 0.75) and overall recommendation (4.08 ± 0.51 and 4.00 ± 0.85). There were no statistical differences between the five models in terms of safety and personalization (*p* > 0.05). For overall recommendation, Med-Diet and DeepSeek-R1 were not significantly different (*p* > 0.05), but both were superior to GLM-Z1-32B (3.50 ± 0.67, *p* < 0.05) and GPT-4o (3.25 ± 0.62, *p* < 0.05). No significant differences were found among GLM-Z1-32B, GPT-4o, and Llama-3.3-70B in most dimensions.

Due to the very small number of rare disease cases (*n* = 3), these findings are exploratory and should not be interpreted as evidence of superior long-tail clinical knowledge. Larger studies are needed to confirm the relative performance of models in rare conditions.

#### 3.3.3 Performance on complex cases

For complex cases involving multiple comorbidities with potentially conflicting nutritional requirements, Med-Diet achieved a mean score of 3.94 ± 0.51, followed by DeepSeek-R1 (3.69 ± 0.39), GLM-Z1-32B (3.63 ± 0.47), GPT-4o (3.41 ± 0.56), and Llama-3.3-70B (3.30 ± 0.43). The LMM revealed a significant overall model effect (*F* (4, 107) = 9.55, *p* < 0.05). Med-Diet significantly outperformed GPT-4o (*p* < 0.05) and Llama-3.3-70B (*p* < 0.05), but its differences from DeepSeek-R1 (*p* > 0.05) and GLM-Z1-32B (*p* > 0.05) did not reach statistical significance. DeepSeek-R1 and GLM-Z1-32B performed similarly (*p* > 0.05), and both were significantly better than Llama-3.3-70B (*p* < 0.05). No significant difference was observed between GPT-4o and Llama-3.3-70B (*p* > 0.05) (Figure 4C).

Dimension-specific analyses revealed that Med-Diet maintained good performance in terms of accuracy (4.04 ± 0.67), practicality (4.08 ± 0.65) and overall recommendation (4.04 ± 0.55), significantly outperforming GPT-4o and Llama-3.3-70B (adjusted *p* < 0.05) (Figure 5C). However, Med-Diet did not differ significantly from DeepSeek-R1 in any dimension (all *p* > 0.05). Notably, GLM-Z1-32B performed competitively in practicality (4.00 ± 0.78), not significantly different from Med-Diet (4.08 ± 0.65, *p* > 0.05). This did not translate to superior overall performance due to deficiencies in other dimensions. In personalization, no pairwise comparisons reached statistical significance. GPT-4o and Llama-3.3-70B consistently scored lower, though GPT-4o was not significantly different from DeepSeek-R1 or GLM-Z1-32B in safety and practicality (adjusted *p* > 0.05). This indicates their difficulty in handling complex scenarios involving multiple coexisting diseases and overlapping dietary restrictions. Compared with general models (which showed a significant performance decline across various dimensions), Med-Diet maintained advantages over weaker general models, but its superiority over DeepSeek-R1 was lost.

Given the limited sample size (*n* = 6) and the heterogeneity of the multimorbidity pattern, the results are also exploratory and need to be validated in larger cohorts.

### 3.4 Model performance across different clinical systems

We further analyzed model performance across seven clinical specialties (cardiology, nephrology, endocrinology, hepatology, neurology, rheumatology and hematology) based on common disease cases. Linear mixed models with Tukey adjustment were applied to each specialty separately. The overall model effect was significant in all specialties (all *p* < 0.05). Med-Diet achieved the highest mean scores (Table 2) in all seven clinical systems, ranging from 3.73 ± 0.65 (neurology) to 4.43 ± 0.42 (rheumatology). In cardiology, Med-Diet (4.04 ± 0.84) significantly outperformed all other models (all *p* < 0.05). DeepSeek-R1 (3.48 ± 0.67) was superior to GLM-Z1-32B (2.74 ± 0.68, *p* < 0.05) and GPT-4o (2.94 ± 0.62, *p* < 0.05). In endocrinology, nephrology, neurology and rheumatology, Med-Diet also showed consistent superiority (all *p* < 0.05 vs. other models). In hepatology, Med-Diet (4.40 ± 0.30) and GLM-Z1-32B (4.17 ± 0.47) performed similarly (*p* > 0.05), and both significantly outperformed GPT-4o and Llama-3.3-70B (*p* < 0.05). DeepSeek-R1 (3.96 ± 0.67) was not significantly different from Med-Diet (*p* > 0.05) or GLM-Z1-32B (*p* > 0.05). In hematology, Med-Diet (3.86 ± 0.68) significantly outperformed GLM-Z1-32B and Llama-3.3-70B (*p* < 0.05), but differences with DeepSeek-R1 (*p* = 0.130) and GPT-4o (*p* = 0.073) were not significant.

**Table 2:** Model performance across different clinical systems.

Clinical system	Med‑Diet	DeepSeek‑R1	GPT‑4o	GLM‑Z1‑32B	Llama‑3.3‑70B
Cardiology	4.04 ± 0.84	3.48 ± 0.67***	2.94 ± 0.62****	2.74 ± 0.68***	3.00 ± 0.69***
Nephrology	4.17 ± 0.81	3.39 ± 0.65***	3.43 ± 0.66***	3.33 ± 0.72***	3.04 ± 0.65***
Endocrinology	4.17 ± 0.72	3.68 ± 0.65***	3.37 ± 0.65***	3.39 ± 0.74***	3.09 ± 0.68***
Hepatology	4.40 ± 0.30	3.96 ± 0.67 ns	3.31 ± 0.59***	4.17 ± 0.47 ns	2.90 ± 0.63***
Neurology	3.73 ± 0.65	3.28 ± 0.68**	3.12 ± 0.67***	3.15 ± 0.70**	2.91 ± 0.66***
Rheumatology	4.43 ± 0.42	3.90 ± 0.52***	3.62 ± 0.48***	3.47 ± 0.54***	2.99 ± 0.48***
Hematology	3.86 ± 0.68	3.34 ± 0.660.52***	3.17 ± 0.64 ns	3.10 ± 0.69***	2.93 ± 0.67***

Data are presented as mean ± standard deviation. * indicates that the mean score of the Mediterranean diet is statistically significantly higher than the score of the corresponding clinical specialty model (linear mixed model, Tukey adjustment, *p* < 0.05). ns indicates that the difference is not statistically significant (*p* ≥ 0.05).

### 3.5 Nutritionist-independent generation vs. model-assisted generation

**Figure 6:**
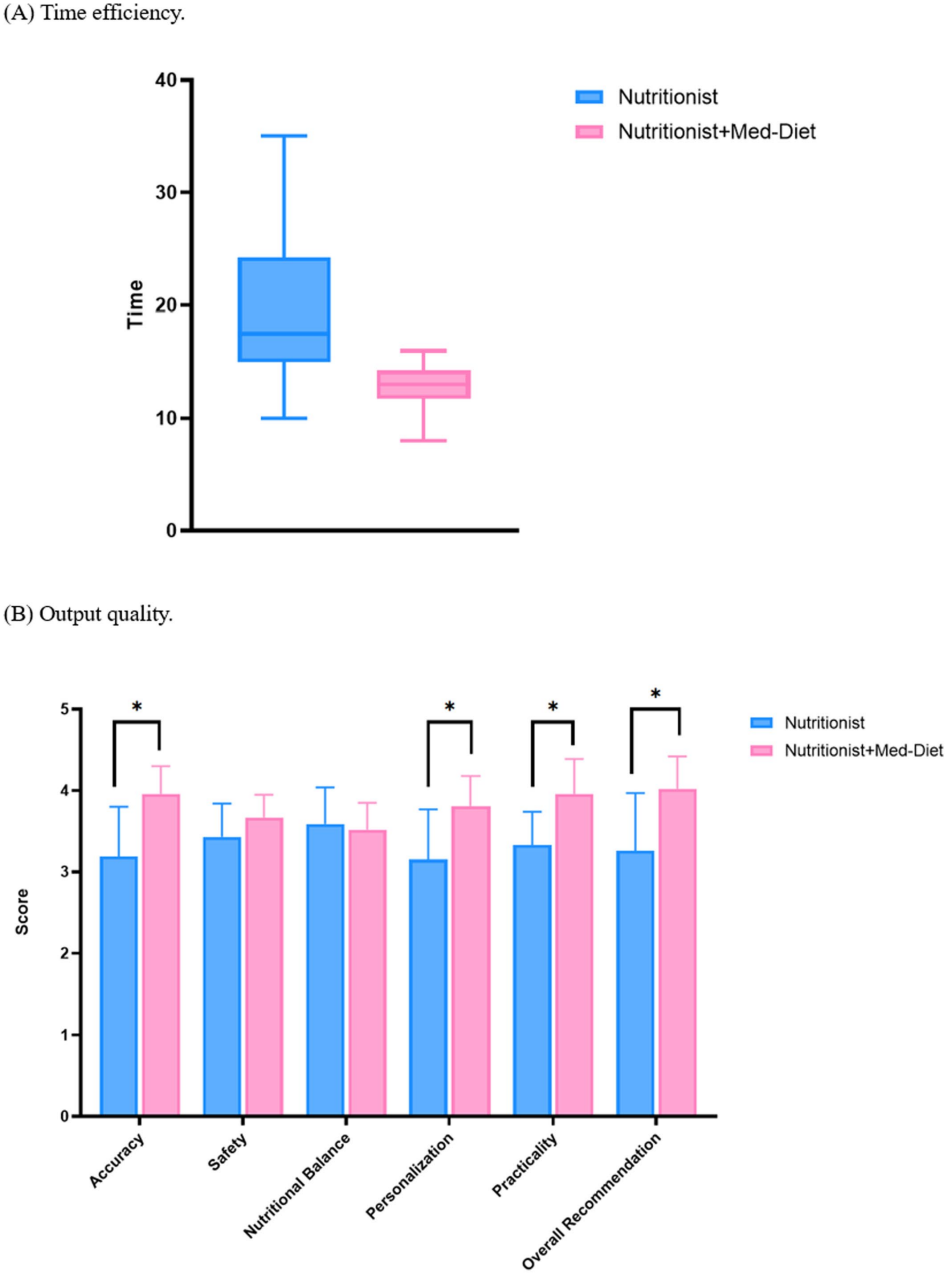
Efficiency and quality comparison between nutritionist-independent and model-assisted dietary plan generation. **(A)** Time efficiency: boxplots of generation time (minutes) for Group A (nutritionist) and Group B (nutritionist + Med-Diet). Paired Wilcoxon signed-rank test: *p* = 0.00031, effect size *r* = 0.85. **(B)** Output quality: mean scores (± SD) for six dimensions evaluated by three experts. Paired Wilcoxon tests with Bonferroni correction (*α* = 0.0083). Asterisks indicate adjusted *p* < 0.05.

Regarding time efficiency, nutritionists independently generating dietary plans (Group A) required a mean of 20.00 ± 7.08 min (median 17.5 min, IQR 8.25 min), whereas those refining Med-Diet drafts (Group B) required 12.83 ± 2.09 min (median 13.00 min, IQR 2.00 min). Group B exhibited a smaller IQR, indicating less variability among the middle 50% of observations and greater consistency in completion times. The overall distribution range was narrow, and the degree of dispersion was small, indicating that the differences in time data between samples in this group were small (Figure 6A). A paired Wilcoxon test showed a statistically significant difference (*p* < 0.05, effect size *r* = 0.85). Given that Group B worked from a Med-Diet draft, the time reduction partly reflects the study design.

For dietary plan quality, paired Wilcoxon tests were performed for each of the six dimensions with Bonferroni correction (*α* = 0.0083). The results showed that accuracy (Group A 3.19 ± 0.61 vs. Group B 3.96 ± 0.34, adjusted *p* = 0.003), personalization (3.15 ± 0.62 vs. 3.81 ± 0.37, adjusted *p* = 0.004), practicality (3.33 ± 0.41 vs. 3.96 ± 0.43, adjusted *p* = 0.008), and overall recommendation (3.26 ± 0.71 vs. 4.02 ± 0.40, adjusted *p* = 0.007) were significantly improved with Med-Diet assistance, all with large effect sizes (*r* > 0.76). No significant differences were observed for safety (3.43 ± 0.41 vs. 3.67 ± 0.28, raw *p* = 0.042, adjusted *p* = 0.250) or nutritional balance (3.59 ± 0.45 vs. 3.52 ± 0.33, *p* > 0.05) (Figure 6B). These preliminary findings suggest that AI’s assistance can lead to higher expert scores for nutritionists’ dietary plans, particularly in accuracy, personalization and practicality. However, these preliminary findings should be viewed as a feasibility signal rather than definitive evidence. These conclusions require validated in larger studies that are closer to clinical workflows.

## 4 Discussion

### 4.1 Inter-rater reliability

Inter-rater reliability was low. The low ICC values indicate limited absolute agreement among raters, reflecting measurement noise and heterogeneity in expert judgment. However, several factors may explain this low ICC. First, our expert panel included physicians from seven different specialties and clinical nutritionists, who have different perspectives on what constitutes an optimal dietary plan, especially for subjective dimensions such as personalization and practicality. Second, the evaluation dimensions themselves are inherently subjective; there is no absolute gold standard for “nutritional balance” or “personalization.” Third, we did not provide rater training or detailed scoring anchors, which likely increased random disagreement. Because all models were evaluated under identical conditions, this random error does not systematically favor any single model. This noise reduces statistical precision but does not distort the comparative ranking among models. More specifically, low ICC makes statistical inference more conservative, not less reliable. Random error inflates standard errors and reduces statistical power, meaning that if a significant difference is still detected under these conditions (e.g., Med-Diet’s consistent superiority in common diseases), that difference is likely to exist in reality, and its effect size may be underestimated. If no significant difference is detected (e.g., Med-Diet vs. DeepSeek-R1 in rare and complex cases), it should be interpreted as “failure to confirm a difference given the current reliability and sample size,” rather than equivalence. In summary, these considerations constrain the core conclusions of this study to comparative expert preference. Low ICC does not undermine the validity of the core conclusions but suggests that future studies should complement subjective ratings with more objective quantitative metrics (e.g., automated guideline concordance scoring, nutrient error analysis). Nonetheless, future research should also consider rater training, more detailed scoring anchors, or objective checklist-based assessments to improve inter-rater reliability.

### 4.2 Overall performance and advantages of the Med-Diet

In this expert-rating study, Med-Diet—an LLM-based agent enhanced with clinical dietary guidelines and structured output constraints—received higher preference scores from clinical experts compared to four general-purpose LLMs (DeepSeek-R1, GPT-4o, GLM-Z1-32B, and Llama-3.3-70B) in this exploratory analysis. Med-Diet achieved superior expert-rated scores in accuracy, safety, nutritional balance, personalization, and practicality. While DeepSeek-R1, as the base model of Med-Diet, demonstrated strong logical reasoning capabilities and even comparable performance to Med-Diet in rare disease scenarios, it exhibited limitations in safety assurance and personalization for common and complex cases. Additionally, in a feasibility experiment, nutritionists completed dietary plans more quickly when refining Med-Diet-generated drafts compared to creating plans *de novo*, though this time difference reflects the study design rather than proven efficiency gains in clinical practice. Additionally, the generated dietary plans received higher expert scores. However, due to the lack of ablation experiments and objective validation, we cannot determine which components of Med-Diet (knowledge injection, structured output templates, or two-stage inference flow) drive performance improvements. Therefore, the finding we observed should be interpreted as preliminary.

### 4.3 Performance across clinical scenarios

The evaluation in this study reveals the comprehensive and robust performance advantages of Med-Diet across different disease types and clinical specialty systems, while also highlighting the limitations of general-purpose large language models (LLMs) in specific medical domains.

For common diseases (e.g., coronary heart disease, diabetes, chronic kidney disease) within specialty systems with well-established nutritional guidelines (e.g., cardiovascular, renal, endocrine systems), Med-Diet demonstrated the most outstanding performance, particularly in accuracy, safety, and practicality, all of which were unanimously recognized by experts. This indicates that experts believe that the dietary plans developed by Med-Diet not only comply with clinical dietary guidelines but also fully consider patients’ daily habits. In contrast, other models showed deficiencies in dimensions such as safety. All models scored relatively low on nutritional balance, indicating that generating diverse, well-balanced daily menus under disease-specific constraints remains challenging.

Results from rare diseases and complex cases scenarios should only be used as exploratory results due to small sample sizes. In rare diseases, Med-Diet and DeepSeek-R1 performed similarly, both outperforming the other models. Notably, in practicality, DeepSeek-R1 even achieved the highest numerical score, though not significantly different from Med-Diet. This suggests that for rare conditions, the base model’s general reasoning capabilities may be sufficient, and additional domain knowledge does not confer a measurable benefit. GPT-4o and Llama-3.3-70B showed marked declines, suggesting that general-purpose models may have limitations in data-sparse domains. However, these results do not support conclusions about long-tail clinical knowledge mastery or superiority in rare disease management and need to be confirmed in larger rare disease cohorts. Future work should focus on few-shot learning from case reports or retrieval-augmented generation from the biomedical literature to improve performance in data-sparse domains. Similarly, in complex cases, Med-Diet’s advantage over its base model DeepSeek-R1 disappeared in all dimensions (all *p* > 0.05), suggesting that when multiple conflicting dietary requirements coexist, the structured knowledge injected into Med-Diet may not fully capture the nuanced trade-offs required. Med-Diet appeared to handle conflicting nutritional needs better than other generic models, indicating that clinicians still preferred Med-Diet-generated plans overall. Notably, GLM-Z1-32B performed competitively in practicality and nutritional balance, which may reflect its training based on diverse real-world data, earning high scores from experts. However, these observations come from small convenience samples. No definitive conclusions can be drawn regarding multimorbidity management performance or the ability to coordinate competing dietary needs in complex clinical scenarios.

The cross-specialty analysis revealed substantial heterogeneity in the performance of general-purpose LLMs. While Med-Diet consistently achieved the highest raw mean scores across all seven specialties, the relative performance of other models varied considerably. In hepatology, GLM-Z1-32B performed unexpectedly well, achieving a mean score close to Med-Diet, with no significant difference. This reflects that GLM-Z1-32B generated dietary plans were more preferred by hepatologists. Due to the small number of cases included in this experiment, it is not possible to conclude that “the training data of this model contains a large number of nutrients related to hepatology, or the dietary principles of liver disease conflicted with general knowledge.” In contrast, in cardiology and nephrology, where nutritional guidelines are strict and evidence-based (e.g., low-sodium, low-potassium diets), GLM-Z1-32B and GPT-4o showed poor performance. In hematology, DeepSeek-R1 and GPT-4o were not significantly different from Med-Diet in overall score, indicating that for certain specialties, the base model (DeepSeek--R1) may already provide acceptable performance without domain adaptation. In neurology, all models scored relatively lower, suggesting that nutritional management for neurological diseases may be particularly challenging for both domain-adapted and general models. These observations suggest that cross-specialty generalization of general-purpose LLMs is uneven, and validation in the target specialty is advisable before deployment. However, these analyses are based on common disease subgroups and vary in sample size across specialties, and the results should be considered exploratory.

### 4.4 Implications for clinical practice

To preliminarily assess the feasibility of Med-Diet as an assistive tool, we conducted a small exploratory experiment. The results show that the median time savings in Group B was 4.5 min (approximately 26% reduction) in terms of time efficiency. This suggested that the time saved allowed nutritionists to manage more patients or allocate more time to complex cases. When dealing with a specific case, AI-generated dietary plan drafts can save nutritionists the time-consuming process of manually finding information and repeating calculations. In terms of dietary plan quality, the four dimensions of accuracy, personalization, practicality, and overall recommendation improved significantly after Bonferroni correction (all adjusted *p* < 0.0083). This indicated that AI-assisted generated dietary plans were recognized by experts for being evidence-based and patient-centric. However, these findings should be viewed as a feasibility signal only, not as strong evidence that Med-Diet improves nutritionist performance in general practice. Larger, multi-center randomized controlled trials that mimic real-world workflows are needed to rigorously evaluate the actual impact of Med-Diet on nutritionist performance.

Future research can further optimize AI algorithms to strengthen their assistance in safety and nutritional balance, and explore how to better combine the professional knowledge of nutritionists with the technical advantages of AI to provide patients with higher-quality nutritional services.

### 4.5 The safety and reliability of the med-diet

In this study, Med-Diet-generated dietary plans received higher expert scores on the safety dimension than general-purpose models. We observed that GPT-4o and Llama-3.3-70B occasionally generated suggestions that seem reasonable but violate guidelines. For example, GPT-4o generated foods with high iodine content such as kelp soup for patients with hyperthyroidism; Llama-3.3-70B will produce foods with high levels of purines in seafood for gout patients. The meal plan generated by DeepSeek-R1 and Llama-3.3-70B is erratic in terms of caloric intake, occasionally providing inappropriately high or low calories for the patient. We also observed that GLM-Z1-32B would falsely quote and fabricate non-existent dietary regimens or principles (Supplementary material S5). Previous studies have noted limitations of general LLMs in nutritional guidance. Concern about the accuracy of diets provided by ChatGPT was raised by a study assessing 56 diets created by ChatGPT for hypothetical individuals with food allergies due to the findings of inaccuracies, with errors involving portions or calories of food, meals, or whole diets (15). Moreover, Qarajeh et al. (9) noted in their study targeting hemodialysis patients that ChatGPT-recommended menus contained high-potassium fruits constituting up to 28% of the 24-h dietary plan, significantly exceeding the upper limit set by nephrology guidelines (<10%). Despite this, the model still generated explanatory text stating “this menu is suitable for kidney patients”. We also noticed that GPT-4o tended to recommend Western-style dishes (e.g., pasta, salmon) in some cases, and Llama-3.3-70B produced English dietary plans. This possibly reflected geographical bias in its training data. However, this observation is based on a few cases and therefore cannot serve as conclusive evidence. Future work could address this through cross-cultural data balancing or local fine-tuning.

### 4.6 Limitations

This study has several limitations. Firstly, the study’s sample size, though covering 17 diseases and 79 cases, remained limited and may not fully capture the diversity of all chronic noncommunicable diseases. Due to the extremely small sample size of rare and complex cases, these findings can only be used as exploratory results. These results cannot prove its superiority in long-tail clinical knowledge mastery and management of multiple chronic diseases. Secondly, the study relied on subjective expert ratings and lacked objective clinical or nutritional validation. We did not perform rule-based guideline concordance analysis (e.g., automated checking of dietary recommendations against clinical practice guidelines), independent adjudication of contraindications (e.g., systematic detection of food-drug interactions or disease-specific forbidden foods), or objective nutritional verification (e.g., comparison of generated nutrient profiles against guideline-recommended targets). Consequently, these findings should not be interpreted as evidence of clinical validity, safety, or reliability. Thirdly, for small-scale human-AI collaboration experiments, this exploratory experiment was intended only as a feasibility signal, not a confirmatory test. Potential biases included small sample sizes, lack of blinding, and possible learning effects despite a one-week separation. Lastly, Med-Diet’s current design is primarily oriented toward plan generation based on static clinical inputs, and it lacks capabilities for interactive, longitudinal dietary guidance—such as real-time feedback on patient adherence, adaptive dietary adjustments based on changes in biochemical markers, or ongoing behavioral support—which are essential for sustained nutritional management in chronic disease care.

## 5 Conclusion

In summary, this expert-rating study demonstrates that Med-Diet-generated dietary plans received higher preference scores from clinical experts compared to general-purpose LLMs. The integration of domain-specific knowledge and structured prompting frameworks appears to improve expert-perceived accuracy and practicality of AI-generated dietary plans. These promising results suggest that Med-Diet holds potential as an assistive tool in clinical nutrition workflows. Future prospective clinical studies are warranted to further validate the safety, efficacy, and real-world impact of Med-Diet on patient outcomes in chronic disease nutrition management.

## Data Availability

Due to ethical restrictions concerning patient privacy, the raw data from this study are not publicly available. However, de-identified data supporting the conclusions may be obtained from the corresponding author upon reasonable request, subject to approval by the institutional ethics committee (Medical Ethics Committee of Qilu Hospital of Shandong University).
