# Supplementary material for: Med-Diet: evaluation of an LLM-based system for clinically guided nutrition care in chronic diseases
Source: Front Nutr. 2026 Jun 12;13:1826469. doi: 10.3389/fnut.2026.1826469 (PMC13303124; doi:10.3389/fnut.2026.1826469)
Supplement: Supplementary file 1 [file Table_1.DOCX]

**Supplementary Material**

Title: Med-Diet: Evaluation of an LLM-Based System for Clinically Guided Nutrition Care in Chronic Diseases

First Author: Yanan Wang, Miaomiao Cheng and Qi Zhang

**Supplementary Material S1.** Structured Prompt Designs for the Med-Diet.

(A) Disease Diagnosis Prompt.

(where document is basic information, diagnoses are diagnostic criteria):

multi_grade_prompt = ChatPromptTemplate.from_messages([

("system", """

You are a clinical expert who needs to output whether the patient has the following diseases or belongs to the following regions based on patient information and disease diagnostic criteria:

"""),

("human", """Patient Information:\n{document}\n\nDiseases/Regions to Determine and Criteria:\n{diagnoses}

Strictly adhere to the following requirements:

1. Return "Yes" for diseases already explicitly diagnosed in the input text

2. Return results only in JSON format, no explanations

3. Output only "Yes" or "No" for each disease determination

4. Output 'No' when unable to determine the patient's region

5. Strictly maintain the output structure unchanged

Format as follows:

{{

"Diabetes": "Yes/No",

"Gout": "Yes/No",

"Obesity": "Yes/No",

"Hyperthyroidism": "Yes/No"

……}}""")

])

(B) Dietary Advice Prompt.

(where diseases are the diseases determined in step 1, nutrition_advice is the dietary advice corresponding to the diseases, document is the patient's basic information):

# Dietary Prescription System Prompt

diet_system = """As a clinical nutritionist, please provide a dietary plan for the patient,

generate content in the following order:

I. Dietary Recommendations

Summarize 3-5 overall dietary points, with line breaks between each point. (Require integration of dietary requirements for all related diseases)

II. Detailed Daily Dietary Plan

1. Generate recipes fully considering the patient's personalized situation (such as age, BMI, dietary preferences, regional preferences, etc.)

2. Ingredients and weights (g/ml) required for each meal, provide weights (g/ml) of required seasonings

3. Consider diverse ingredient combinations, conforming to Chinese dietary habits; if dietary recommendations conflict with each other, weigh pros and cons for comprehensive recommendations

4. Total calories and content/proportion of three major nutrients for each meal

5. Dietary plan includes breakfast, lunch, dinner, and snacks. Provide alternative foods when necessary (such as staple foods or vegetables, etc.)

III. Precautions/Reminders

1. List the most critical liquids/micronutrients the patient must consume daily but need strict intake limits (e.g., daily liquid intake <1000ml)

2. List dangerous foods for this patient; consuming these foods may worsen the condition (e.g., gout patients: reduce intake of animal organs, seafood and other high-purine foods)

3. Remind patients of indicators they should monitor

4. For multiple restrictions, dangerous foods, or monitoring indicators, output in "1. XXX\n2. XXX ……" format

Return results only in JSON format, no explanations. Generate format as follows:

{{

"Dietary Recommendations": "1. XXX\n2. XXX \n3. XXX \n4. XXX \n5. XXX ",

"Detailed Dietary Plan": {{

"Breakfast": "Meal name (specific foods and doses)\nTotal calories: XXXkcal, Protein XXg(XX%), Fat XXg(XX%), Carbohydrates XXg(XX%)",

"Lunch": "Meal name (specific foods and doses)\nTotal calories: XXXkcal, Protein XXg(XX%), Fat XXg(XX%), Carbohydrates XXg(XX%)",

"Dinner": "Meal name (specific foods and doses)\nTotal calories: XXXkcal, Protein XXg(XX%), Fat XXg(XX%), Carbohydrates XXg(XX%)",

"Snack": "Meal name (specific foods and doses)\nTotal calories: XXXkcal, Protein XXg(XX%), Fat XXg(XX%), Carbohydrates XXg(XX%)"

}},

"Restrictions": "XXX",

"Dangerous Foods": "XXX",

"Monitoring Indicators": "XXX"

}}

"""

diet_prompt = ChatPromptTemplate.from_messages([

("system", diet_system),

("human", """Please develop a dietary plan for the patient based on the following information:

1. Patient's diseases/regions: {diseases}

2. Nutritional advice: {nutrition_advice}

3. Patient basic information: {document}

Please generate a dietary plan including overall points and detailed plan:""")

])

**Supplementary Table S2:** Evaluation Form.

Each question was evaluated with a score ranging from 1 to 5.

1= Completely inconsistent

2= Inconsistent

3= Neutral

4= Consistent

5= Fully Consistent

1. Common Diseases and Complex Cases.

| **Evaluation Dimension** | **Score** |
| --- | --- |
| **Accuracy** |  |
| Do the core nutritional principles align with current guidelines/standards for this disease? |  |
| Does it help manage key indicators of this disease? |  |
| **Safety** |  |
| Does it effectively avoid foods or nutrients that may worsen the disease state? |  |
| Does it include necessary precautions or reminders (e.g., “This recipe is an example and needs confirmation by a doctor/nutritionist,” “Those strictly limiting potassium need to be cautious of XX ingredients”)? |  |
| **Nutritional Balance** |  |
| Does it include all essential nutrients? |  |
| Are the provided total calories and content of various nutrients adequate and proportionally reasonable? |  |
| **Personalization** |  |
| Does it fully consider the patient’s individual characteristics (BMI, energy requirements, abnormal lab indicators, etc.)? |  |
| **Practicality** |  |
| Is the recipe content (food types, portions) described clearly, specifically, and unambiguously? |  |
| **Overall Recommendation** |  |
| To what extent are you willing to recommend this recipe? |  |

1. Rare Diseases.

| **Evaluation Dimension** | **Score** |
| --- | --- |
| **Accuracy** |  |
| Do the core nutritional principles align with current guidelines/standards for this disease? |  |
| Does it strictly adhere to the precise control requirements for key restricted nutrients specific to this rare disease? |  |
| Does it help manage key indicators of this disease? |  |
| **Safety** |  |
| Does it include necessary precautions or reminders (e.g., "This recipe is an example and needs confirmation by a doctor/nutritionist," "PKU patients need to be cautious of XX ingredients")? |  |
| **Nutritional Balance** |  |
| While strictly restricting specific nutrients, does it include other essential nutrients? |  |
| Are the provided total calories and content of various nutrients adequate and proportionally reasonable? |  |
| Is the necessity and usage method of Formulas for Special Medical Purposes (e.g., no/low phenylalanine protein powder, specific carbohydrate sources) clearly and correctly provided? |  |
| **Personalization** |  |
| Does it fully consider the patient's individual characteristics (BMI, energy requirements, abnormal lab indicators, etc.)? |  |
| **Practicality** |  |
| Is the recipe content (food types, portions, usage instructions for special formulas, etc.) described clearly, specifically, and unambiguously? |  |
| **Overall Recommendation** |  |
| To what extent are you willing to recommend this recipe? |  |

1. Nutritionist Group vs. Nutritionist + Med-Diet Group.

| **Evaluation Dimension** | **Score** |
| --- | --- |
| **Accuracy** |  |
| Do the core nutritional principles align with current guidelines/standards for this disease? |  |
| **Safety** |  |
| Does it avoid situations that could exacerbate the patient's condition (including factors such as the control of disease-related nutrients, allergenic foods, and drug-food interactions)? |  |
| **Nutritional Balance** |  |
| Are the provided total calories and content of various nutrients adequate and proportionally reasonable? Is the distribution of meals and energy throughout the day appropriate? |  |
| **Personalization** |  |
| Does it fully consider the patient's disease status, clinical lab indicators, as well as personal preferences or cultural background? |  |
| **Practicality** |  |
| Is the recipe content described clearly, specifically, and unambiguously? Are the ingredients easy to obtain and are the cooking methods simple and feasible? |  |
| **Overall Recommendation** |  |
| To what extent are you willing to recommend this recipe? |  |

**Supplementary Table S3. Department‑specific ICCs.**

| **Department** | **ICC (95% CI)** |
| --- | --- |
| Cardiology | 0.36 (0.26–0.45) |
| Nephrology | 0.28(0.23–0.30) |
| Endocrinology | 0.27 (0.22–0.33) |
| Hepatology | 0.37 (0.28–0.46) |
| Neurology | 0.14 (0.07–0.22) |
| Rheumatology | 0.25 (0.17–0.34) |
| Hematology | 0.33 (0.24–0.42) |

**Supplementary Table S4. Sensitivity analysis using ordinal mixed‑effects models.**

| **Dimension** | **Comparison** | **Estimate** | **SE** | **z-ratio** | **p-value** |
| --- | --- | --- | --- | --- | --- |
| **Accuracy** | Med‑Diet vs DeepSeek‑R1 | -1.80 | 0.21 | -8.78 | <0.05 |
|  | Med‑Diet vs GPT‑4o | -2.78 | 0.21 | -12.97 | <0.05 |
|  | Med‑Diet vs GLM‑Z1‑32B | -2.56 | 0.22 | -11.80 | <0.05 |
|  | Med‑Diet vs Llama‑3.3‑70B | -3.81 | 0.23 | -16.83 | <0.05 |
| **Safety** | Med‑Diet vs DeepSeek‑R1 | -1.87 | 0.19 | -9.83 | <0.05 |
|  | Med‑Diet vs GPT‑4o | -2.09 | 0.19 | -10.88 | <0.05 |
|  | Med‑Diet vs GLM‑Z1‑32B | -2.51 | 0.20 | -12.55 | <0.05 |
|  | Med‑Diet vs Llama‑3.3‑70B | -3.38 | 0.21 | -16.20 | <0.05 |
| **Nutritional Balance** | Med‑Diet vs DeepSeek‑R1 | -1.09 | 0.19 | -5.83 | <0.05 |
|  | Med‑Diet vs GPT‑4o | -2.07 | 0.20 | -10.51 | <0.05 |
|  | Med‑Diet vs GLM‑Z1‑32B | -1.79 | 0.20 | -9.13 | <0.05 |
|  | Med‑Diet vs Llama‑3.3‑70B | -2.88 | 0.21 | -13.69 | <0.05 |
| **Personalization** | Med‑Diet vs DeepSeek‑R1 | -1.10 | 0.20 | -5.56 | <0.05 |
|  | Med‑Diet vs GPT‑4o | -1.97 | 0.20 | -9.63 | <0.05 |
|  | Med‑Diet vs GLM‑Z1‑32B | -1.60 | 0.20 | -7.87 | <0.05 |
|  | Med‑Diet vs Llama‑3.3‑70B | -2.66 | 0.22 | -12.41 | <0.05 |
| **Practicality** | Med‑Diet vs DeepSeek‑R1 | -1.47 | 0.20 | -7.23 | <0.05 |
|  | Med‑Diet vs GPT‑4o | -2.31 | 0.21 | -10.89 | <0.05 |
|  | Med‑Diet vs GLM‑Z1‑32B | -2.26 | 0.22 | -10.48 | <0.05 |
|  | Med‑Diet vs Llama‑3.3‑70B | -3.14 | 0.22 | -14.12 | <0.05 |
| **Overall Recommendation** | Med‑Diet vs DeepSeek‑R1 | -2.45 | 0.30 | -8.05 | <0.05 |
|  | Med‑Diet vs GPT‑4o | -3.56 | 0.25 | -14.03 | <0.05 |
|  | Med‑Diet vs GLM‑Z1‑32B | -3.64 | 0.26 | -14.22 | <0.05 |
|  | Med‑Diet vs Llama‑3.3‑70B | -4.85 | 0.27 | -17.80 | <0.05 |

**Supplementary Material S5. Failure or Error Cases.**

**DeepSeek-R1: When generating dietary plans, the patient's specific situation (age, BMI, activity level, etc.) is not fully taken into account, resulting in low caloric intake.**

**Dietary Plan 1**: ###One-Day Customized Recipe (Low Salt, Kidney Protection, Anti-Inflammatory Orientation)

---

#### **Breakfast: Whole Grain Sandwich + Milk + Fruit**

- **Whole wheat bread** 2 slices (50g)

- **Boiled egg** 1 piece (50g)

- **Lettuce, tomato slices** 2 slices (50g)

- **Low-fat milk** 200ml

- **Apple** half (80g)

**Total calories**: 420 kcal

**Nutrients**

- Protein: 23g (22%)

- Fat: 12g (26%)

- Carbs: 56g (52%)

**Note**:

1. Whole wheat bread provides dietary fiber and stabilizes blood sugar; Milk supplements calcium (prednisone is prone to osteoporosis);

2. Avoid sauces and reduce sodium intake; Apples contain antioxidants (quercetin) to support anti-inflammatory.

---

#### **Lunch: Multigrain rice + steamed sea bass + stir-fried vegetables**

- **Multigrain Rice** 100g (Black Rice + Brown Rice)

- **Steamed sea bass** 100g (remove the scales, use only ginger slices to remove the fishy smell)

- **Garlic broccoli** 150g + **Carrot slices** 50g (5g olive oil for quick stir-fry)

- **Winter melon corn soup** 1 bowl (no salt, umami comes from corn)

**Total calories**: 550 kcal

**Nutrients**

- Protein: 30g (22%)

- Fat: 18g (29%)

- Carbohydrates: 65g (49%)

**Note**:

1. Sea bass high-quality low-fat protein, containing Omega-3 fatty acids, reduces inflammation;

2. Broccoli contains sulfur glycosides, which assist liver metabolism and immunosuppressants; Winter melon diuresis assists in lowering blood pressure.

---

#### **Afternoon Extra: Nut Yogurt Cup**

- **Sugar-free low-fat yogurt** 100g

- **Blueberries** 50g

- **Walnut kernels** 3 pieces (about 10g)

**Total calories**: 150 kcal

**Nutrients**

- Protein: 5g (13%)

- Fat: 10g (60%)

- Carbs: 8g (27%)

**Note**: Walnuts provide α-linolenic acid (anti-inflammatory), and blueberry anthocyanins protect the endothelium of blood vessels.

---

#### **Dinner: Shredded chicken soba + spinach**

- **Soba** 80g (cooked weight)

- **Shredded chicken breast** 60g (boiled torn strips)

- **Cold spinach** 150g (3g sesame oil, garlic vinegar to taste)

- **Nori tofu soup** (50g soft tofu, 1g seaweed)

**Total calories**: 480 kcal

**Nutrients**

- Protein: 28g (24%)

- Fat: 12g (23%)

- Carbohydrates: 65g (53%)

**Remarks**: Soba noodles low GI + high rutin (improve microcirculation); Seaweed supplements selenium and strengthens immune function.

---

#### **Late-night snack (optional): lotus seed white fungus soup**

- **White fungus (dried)** 5g + **lotus seeds** 10 pieces (boiled without sugar)

**Total calories**: 50 kcal

**Efficacy**: White fungus polysaccharides regulate immunity, lotus seeds help sleep and reduce hormone side effects.

---

### **Total Daily Intake**

- **Calories**: 1650 kcal (can be increased or decreased by 10% with activity)

- **Protein**: 86g (21%)

- **Fat**: 52g (28%)

- **Carbohydrates**: 194g (51%)

---

### **Personalized Health Advice**

1. **Salt limit and pressure control**: < 5g of salt throughout the day (change to herbs and lemon juice for flavor), avoid soy sauce and pickles.

2. **Avoid photosensitive foods**: such as celery, amaranth, figs (UV sensitivity exacerbates SLE).

3. **Kidney protection details**: Protein intake is designed according to 0.8g/kg (to avoid increasing the burden on the kidneys).

4. **Drug synergy**: Prednisone may cause hypokalemia, and regular monitoring can increase bananas and oranges (if blood potassium is normal).

5. **Water intake**: 1500ml per day (under the premise of normal urine output, avoid edema).

---

If you need to adjust the taste or notice edema/creatinine abnormalities, please contact your doctor and dietitian in time. "

**GPT-4o**

**1. The resulting recipes contain a Westernized diet, such as grilled salmon, salmon and vegetable salad.**

**Dietary Plan 1:** ### Lunch

- **Food**: Grilled salmon (150g), steamed broccoli (100g), quinoa rice (quinoa 50g, rice 50g), tofu soup

- **Nutrients and Calories**:

- Total calories: about 600 kcal

- Protein: 35g (about 24%)

- Fat: 20g (about 30%)

- Carbohydrates: 70g (about 46%)

**Dietary Plan 2:** ### Lunch

- Salmon and vegetable salad

- 150g grilled salmon

- Leafy greens (e.g., spinach, romaine lettuce)

- Cherry tomatoes

- 1/2 avocado

- Olive oil and lemon juice to taste

- 1 cup brown rice

**2. The dietary plan generated for the patient contains foods that are not recommended or are excessive for the disease.**

**Dietary Plan 3:** ### Lunch:

- Whole wheat rice (brown rice + quinoa mix, 100g uncooked)

- Grilled chicken breast (100g)

- Sautéed carrots with broccoli (150g)

- Tofu kelp soup (50g tofu, 30g kelp)

**Dietary Plan 4:** "Based on patient's health and dietary adjustment needs, especially considering the symptoms of cirrhosis decompensation and glomerulonephritis, I recommend a diet that is low in protein, salt, high in fiber, and rich in vitamins and minerals to help reduce the burden on the liver and kidneys and support overall health. Here is a one-day recipe design:

### Breakfast

- **Oatmeal**: 50g of oats, made into porridge with warm water, add a small amount of goji berries.

- **Steamed pumpkin**: 250g pumpkin, steamed and served.

- **Boiled egg whites**: 1 egg (only the egg whites’ portion).

**Nutritional Analysis**:

- Calories: about 240 kcal

- Protein: 10 g (about 16%)

- Fat: 5 g (about 18%)

- Carbohydrates: 40 g (about 66%)

### Lunch

- **Steamed fish**: 150g sea bass, slightly seasoned with ginger and green onions, avoid too much salt.

- **Spinach and tofu soup**: 100g spinach, 50g tofu, a little salt.

- **Brown rice**: 100g brown rice, cooked.

**Nutritional Analysis**:

- Calories: about 400 kcal

- Protein: 30 g (about 30%)

- Fat: 10 g (about 22%)

- Carbohydrates: 55 g (about 48%)

### Dinner

- **Steamed chicken breast**: 100g chicken breast, steamed, no seasoning.

- **Stir-fried vegetables with broccoli and carrots**: 150g broccoli, 50g carrots, and a small amount of olive oil stir-fried.

- **Mashed sweet potato**: 100g sweet potatoes, steamed and mashed.

**Nutritional Analysis**:

- Calories: about 350 kcal

- Protein: 25 g (about 28%)

- Fat: 8 g (approx. 20%)

- Carbohydrates: 50 g (about 52%)

### Total daily calorie and nutrient analysis

- **Total calories**: about 990 kcal

- **Protein**: 65 g (approx. 24%)

- **Fat**: 23 g (about 20%)

- **Carbohydrates**: 145 g (about 56%)

### Dietary advice and precautions

- **Protein intake**: Due to the decline in liver function, protein is mainly high-quality and low-biovalue protein, and should not be too much to avoid increasing the burden on the kidneys.

- **Low-Salt Diet**: A low-salt diet can help reduce fluid retention, especially in cases of ascites.

- **Quit alcohol**: Continue to abstain from alcohol strictly to avoid further liver damage.

- **Vitamin and mineral supplements**: Consuming more fresh fruits and vegetables may require supplements such as B vitamins and multivitamins.

It is recommended that patient follow up on medical consultations regularly and adjust his diet according to the development of the disease. Attention should be paid to the balance and safety of the diet, and please make personalized adjustments under the guidance of a doctor or dietitian. "

**GLM‑Z1‑32B: The generated dietary plans falsely reference and fabricate non-existent diets.**

**Dietary Plan 1:** #### **Night Extra Meal (5%|80 kcal) **

- **Warm milk + ginger jujube tea**

- 200ml (60kcal) skim milk

- 1g ginger powder + 2 pitted red dates (20kcal, digestive)

- Inositol powder 500mg (tested to lower homocysteine by 5-15%)

**Dietary Plan 2:** This scheme has been validated by the DRIS (Dietary Reference Intakes) verification system.

**Dietary Plan 3:** --- Special nutritional interventions---

1. Coronary heart rate monitoring meal:

200mg omega-3 fatty acids per 2 meals (sea bass + flaxseed oil)

With plasma potassium maintenance: Potassium supplementation package (banana + spinach + zucchini)

2. Muscle-Bridge Protective Diet:

\u2012 7:00 a.m. "Turmeric Milk" (containing 3g curcumin + black pepper)

\u2012 Supplement with 500mg of CoQ10 2 hours after meals (Food source: sunflower seeds)

3. Metabolic memory blockade:

Each meal contains 2g of phenolic acid compounds ((1) green tea extract (2) purple cabbage juice)

**Llama-3.3-70B**

**Dietary Plan 1: The generated dietary plan is in English.**

" generaré una dieta personalizada para el paciente，considerando su condición médica y necesidades nutricionales.

**Objetivos de la dieta:**

1. Controlar el nivel de urea y creatinina en sangre.

2. Mantener un buen estado nutricional y prevenir la malnutrición.

3. Ayudar a controlar la hipertensión y la anemia.

4. Reducir el riesgo de complicaciones relacionadas con la enfermedad renal crónica.

**Recomendaciones generales:**

1. Ingerir una dieta baja en proteínas (0,8-1,2 g/kg/día) para reducir la carga de trabajo en los riñones.

2. Limitar el consumo de sodio a menos de 2 g al día para controlar la hipertensión.

3. Ingerir una dieta rica en frutas, verduras y granos enteros para obtener fibra, vitaminas y minerales.

4. Evitar alimentos ricos en potasio, como plátanos, espinacas y aguacates.

5. Limitar el consumo de líquidos a 1-2 litros al día, dependiendo de la condición del paciente.

**Menú sugerido para un día:**

**Desayuno:**

* Avena cocida con leche descremada y azúcar moreno (250 kcal, 10g de proteínas, 40g de carbohidratos, 10g de grasas)

* Una fruta (manzana o pera) (50 kcal, 0,5g de proteínas, 15g de carbohidratos, 0,5g de grasas)

**Almuerzo:**

* Pollo a la plancha con arroz integral y verduras al vapor (400 kcal, 30g de proteínas, 40g de carbohidratos, 15g de grasas)

* Ensalada de lechuga, tomate y pepino con aceite de oliva y vinagre (100 kcal, 5g de proteínas, 10g de carbohidratos, 10g de grasas)

**Merienda:**

* Yogur natural bajo en grasa con miel y frutas secas (150 kcal, 10g de proteínas, 30g de carbohidratos, 5g de grasas)

**Cena:**

* Pescado al horno con quinoa y brócoli al vapor (500 kcal, 35g de proteínas, 40g de carbohidratos, 20g de grasas)

**Snack nocturno:**

* Una taza de té herbal con un poco de azúcar moreno (50 kcal, 0,5g de proteínas, 15g de carbohidratos, 0,5g de grasas)

**Total diario:**

* Calorías: 1450 kcal

* Proteínas: 85g (12% del total de calorías)

* Carbohidratos: 235g (55% del total de calorías)

* Grasas: 65g (33% del total de calorías)

**Notas:**

* Es importante que el paciente consulte con un nutricionista o un profesional de la salud para obtener una dieta personalizada y ajustada a sus necesidades individuales.

* La dieta debe ser revisada y ajustada regularmente para asegurarse de que esté cumpliendo con los objetivos de tratamiento y las necesidades nutricionales del paciente."

**Dietary Plan 2: The calories produced for older men with normal BMI are higher.**

According to Patient's individual situation, the daily recipe is generated as follows:
**Breakfast**
* Food: oatmeal with milk, bananas, walnuts
* Serving size: 250ml oatmeal, 100ml milk, 1 banana, 10g walnuts
* Total calories: 420 kcal
* Three major nutrient contents: + Protein: 20g (15%)
+ Fat: 15g (32%)
+ Carbohydrates: 60g (53% of the total)
 **Lunch**
* Food: Lean meat (chicken) steamed cooker with vegetables (broccoli, carrots), brown rice
* Serving size: 100g chicken, 100g broccoli, 50g carrot, 150g brown rice
* Total calories: 540 kcal
* Three major nutrient contents: + Protein: 35g (23%)
+ Fat: 10g (17%)
+ Carbohydrates: 70g (60%)
**Dinner**
* Food: Fish (cod) steamed cooker with vegetables (spinach, sweet potato), whole grain bread
* Serving size: 100g cod, 100g spinach, 50g sweet potato, 50g whole wheat bread
* Total calories: 460 kcal
* Three major nutrient contents: + Protein: 30g (22%)
+ Fat: 15g (29%)
+ Carbohydrates: 55g (49%)
**Snacks**
* Food: apples, almonds
* Serving size: 1 apple, 10g almond
* Total calories: 150 kcal
* Three major nutrient contents: + Protein: 2g (5%)
+ Fat: 8g (43% of the total) + Carbohydrates: 25g (52%)
**Total daily intake**
* Total calories: 2070 kcal
* Three major nutrient contents: + Protein: 87g (17% of the total) + Fat: 63g (27%)
+ Carbohydrates: 290g (56%)
Considering Patient's chronic kidney disease stage 5 and hypertension grade 3, it is necessary to limit the intake of sodium, potassium, and phosphorus, while ensuring sufficient protein and calorie intake. Therefore, choose foods that are low in sodium, potassium, and phosphorus in the recipe, and add some potassium-rich foods (such as bananas, walnuts) to help correct the low potassium state. In addition, the recipe includes foods rich in omega-3 fatty acids (such as cod) to help lower blood pressure and improve cardiovascular health.

**Dietary Plan 3: The dietary plan generated for the patient contains foods that are not recommended for the disease.**

#### Lunch

- **Grilled chicken breast**: 100g

- **Steamed mung beans**: 100g

- **Steamed sea bass**: 100g

- **Mixed Vegetable Salad**: 200g

- **Total calories**: about 500 kcal

- **Protein**: 40g (30% of total calories)

- **Fat**: 15g (25% of total calories)

- **Carbohydrates**: 40g (35% of total calories)

#### Dinner

- **Steamed shrimp**: 100g

- **Grilled eggplant**: 100g

- **Rice**: 50g

- **Clear soup**: 200ml

- **Total calories**: about 350 kcal

- **Protein**: 20g (20% of total calories)

- **Fat**: 10g (25% of total calories)

- **Carbohydrates**: 40g (45% of total calories)

*Only some representative dietary plans were selected.*
